# Novel Antischistosomal Drug Targets: Identification of Alkaloid Inhibitors of SmTGR via Integrated In Silico Methods

**DOI:** 10.3390/pathogens14060591

**Published:** 2025-06-15

**Authors:** Valéria V. M. Paixão, Yria J. A. Santos, Adriana O. Fernandes, Elaine S. Conceição, Ricardo P. Rodrigues, Daniela A. Chagas-Paula, Silvio S. Dolabella, Tiago B. Oliveira

**Affiliations:** 1 Posgraduate Program in Chemistry—PPGQ, Federal University of Sergipe, Av. Marcelo Deda Chagas, s/n, Bairro Rosa Elze, São Cristóvão 49107-230, SE, Brazil; valeriavieiramp1994@gmail.com (V.V.M.P.); yria.a.santos@gmail.com (Y.J.A.S.); elainesantosconceicao96@gmail.com (E.S.C.); 2 Postgraduate Program in Biotechnology—PROBIO, Federal University of Sergipe, Av. Marcelo Deda Chagas, s/n, Bairro Rosa Elze, São Cristóvão 49107-230, SE, Brazil; ad00fernandes@gmail.com; 3 Faculty of Pharmaceutical Sciences, University of Campinas, Rua Cândido Portinari, 200, Cidade Universitária, Campinas 13083-871, SP, Brazil; rrodrigues@fcf.unicamp.br; 4 Chemistry Institute, Federal University of Alfenas, Rua Gabriel Monteiro da Silva, Alfenas 37130-001, MG, Brazil; daniela.chagas@unifal-mg.edu.br; 5 Postgraduate Program in Parasite Biology, Federal University of Sergipe, Av. Marcelo Deda Chagas, s/n, Bairro Rosa Elze, São Cristóvão 49107-230, SE, Brazil; 6 Postgraduate Program in Pharmaceutical Sciences, Federal University of Sergipe, Av. Marcelo Deda Chagas, s/n, Bairro Rosa Elze, São Cristóvão 49107-230, SE, Brazil; 7 Pharmacy Department, Federal University of Sergipe, Av. Marcelo Deda Chagas, s/n, Bairro Rosa Elze, São Cristóvão 49107-230, SE, Brazil

**Keywords:** *Schistosoma mansoni*, schistosomiasis, thioredoxin glutathione reductase, QSAR, molecular docking, alkaloids

## Abstract

Schistosomiasis mansoni is a neglected tropical disease caused by the parasite *Schistosoma mansoni*, affecting approximately 200 million people annually. Currently, treatment relies primarily on a single drug, praziquantel (PZQ), which shows limited efficacy against the parasite’s immature forms. As a result, Thioredoxin Glutathione Reductase from *S. mansoni* (SmTGR) has emerged as a promising target for novel drug development. This study presents the development of integrated in silico methods to identify alkaloids from medicinal plants with potential activity against *S. mansoni*. Fourteen alkaloids were identified, with predicted activity ranging from 61.3 to 85.2%. Among these, lindoldhamine and daibucarboline A demonstrated, for the first time, potential SmTGR inhibition, with probabilities of 85.2% and 75.8%, respectively. These findings highlight the potential of these alkaloids as promising candidates for the development of new therapies against schistosomiasis.

## 1. Introduction

Schistosomiasis is a neglected tropical disease caused by trematodes of the genus *Schistosoma* [1,2,3,4]. Six species of *Schistosoma* parasitize humans, including, *Schistosoma japonicum*, *Schistosoma mansoni*, *Schistosoma haematobium*, *Schistosoma guineenses*, *Schistosoma mekongi*, and *Schistosoma intercalatum.* The first three species have global importance, while infections by the other species have local prevalence in their respective endemic regions [5,6,7]. However, the most common species responsible for the disease are the parasites *S. haematobium*, *S. japonicum*, and *S. mansoni*, transmitted by the snails *Bulinus*, *Oncomelania*, and *Bioamphalaria*, respectively [7,8,9]. *S. mansoni* is the primary species responsible for hepatic and intestinal schistosomiasis in Africa and has also been reported in South America [10]. Additionally, this parasite is present in the Caribbean and the Middle East [5]. Brazil has the highest number of cases of *S. mansoni* among Latin American countries, with an estimated 1.5 million people infected by the parasite [11].

The current treatment for schistosomiasis involves the oral administration of a single drug, praziquantel (PZQ), which has been utilized for over 40 years [1,5,12]. PZQ is considered safe and effective against all six species of *Schistosoma* that can infect humans and can cause mild and transient side effects, achieving cure rates of 60 to 70%. However, it lacks efficacy against immature forms of the parasites and does not prevent reinfection in individuals living in endemic areas. Hence, there is an urgent need to discover new schistosomicidal drugs [1,6,7,12,13,14].

Thioredoxin glutathione reductase (TGR), a multifunctional enzyme responsible for the redox metabolism of the parasite, has been highlighted as a promising target for developing new drugs to treat schistosomiasis. TGR acts in the detoxification of reactive oxygen species (ROS) generated by the host’s immune response and the digestion of red blood cells [13,15,16]. In mammalian cells, ROS detoxification relies on two primary systems: the thioredoxin (Trx) pathway and the glutathione (GSH) pathway. The Trx pathway involves (i) NADPH, which donates electrons through two flavoenzymes oxidoreductases; (ii) thioredoxin reductase (TrxR), which accepts reductants from NADPH and transfers them to (iii) Trx, which, in turn, reduces several targets. The GSH pathway includes (i) NADPH and (ii) Glutathione Reductase (GR), which transfers electrons from NADPH to the glutathione disulfide (GSSG), producing two molecules of (iii) reduced glutathione (GSH). GSH transfers electrons to (iv) glutaredoxin (Grx), which reduces several substrates. Both the Trx and GSH systems depend on NADPH for enzymatic reduction. In schistosomes, a single system for ROS detoxification exists: the TGR, which transports electrons from NADPH to Trx, and GSH/GSSG, which combines TrxR and Grx domains [15,17,18,19].

Natural products have played and continue to play an important role in the discovery of new compounds for the treatment of various diseases, including schistosomiasis [20,21,22]. Recent studies on secondary metabolites have highlighted alkaloids as promising candidates for activity against *S. mansoni* [23,24,25]. Furthermore, molecular docking studies have identified TRG as a potential binding target for these alkaloids [8,26].

The structural diversity of alkaloids and their successful therapeutic applications [27] can be further enhanced through the development of in silico approaches aimed at predicting potentially active alkaloids from diverse plant species from large datasets.

Ligand-based (LBVS) and structure-based (SBVS) virtual screening are key strategies employed to identify new hits in chemical libraries [28]. However, in recent years, quantitative structure activity relationship (QSAR) and molecular docking models have gained importance in VS [29,30,31,32,33]. Moreover, the integration of techniques has become prevalent in VS studies, as ligand-based QSAR models complement structure-based approaches, thereby enhancing statistical performance [8,34].

Based on the above, this study developed a methodology for constructing calibrated QSAR models for alkaloid prediction using a chemically diverse database, resulting in models capable of covering a broad chemical space. The developed models were applied to predict the activity of alkaloids from medicinal plants. From the combination of the LBVS and SBVS methods, 14 alkaloids were selected as promising inhibitors of SmTGR. Of these, the alkaloids epiisopiloturine, epiisopilosine, pilosine, and isopilosine, in studies by Rocha et al. [24], showed schistosomicidal activity. In addition, the present study selected 10 alkaloids that have not yet been reported in the literature as active against *S. mansoni*. Among them, it is possible to mention the alkaloids lindoldhamine and daibucarboline A as potential inhibitors of SmTGR. These alkaloids, after experimental validation, may contribute to the development of new drug candidates for the treatment of schistosomiasis.

## 2. Materials and Methods

All statistical calculations in this study were performed using the Knime Analytics Platform v. 4.0.2 (KNIME AG, Zurich, Switzerland) [35].

### 2.1. Dataset Treatment and Balancing (AlcSmTGR)

As input data, chemical structures tested against the SmTGR enzyme were used, obtained from the PubChem Bioassay Database (AID: 485364) [36]. This bank was formed by 356,643 chemical structures of substances that were tested in vitro using high-throughput screening specifically targeting TGR. Initially, 236,656 chemical structures that did not have C, O, N, and H atoms in their molecular structure were removed to maintain substances structurally more similar to alkaloids. After that, organometallics, inorganic compounds, salts, and mixtures were removed, resulting in a dataset comprising 116,978 chemical structures. Sequentially, 5151 chemical structures with inconclusive activity information and structures classified as enzyme activators were excluded. In the analysis of duplicates, 854 substances were removed. In the pan-assay interference compounds (PAINS) identification stage, 7536 highly reactive substances were identified and excluded. At the end of the process, 103,437 substances remained in the database, of which 2843 were active and 100,594 were inactive.

To balance the dataset, the subsampling technique based on Tanimoto similarity was used to reduce the number of samples from the majority class, where only the structures most similar to alkaloids were selected, which reduced the number of chemical structures classified as inactive. In this approach, each entry from the alkaloid structure dataset (the bank of alkaloid chemical structures) was selected, and the reference table (the bank of chemical structures tested in SmTGR) was searched for compounds that met the similarity criteria. Thus, based on the Tanimoto distance [37], each alkaloid was used to search for substances in the library of structures tested in the enzyme that was most similar to them. As a result, the inactive substances most similar to alkaloids were selected from the bank of tested chemical structures for the development of QSAR models. Finally, a structured bank was obtained with 2504 substances tested in vitro, of which 1325 were active and 1179 were inactive.

### 2.2. Calculation and Selection of Molecular Descriptors

In total, 5623 two-dimensional chemical descriptors were calculated, of which 4149 were calculated in AlvaDesc v. 2.0 (Alvascience SRL, La Spezia, Italy) [38], 129 in the RDkit v. 2021.03.1 package (RDKit Community (coordinated by Dr. Greg Landrum), Global (distributed development)) [39], and 1.345 in PaDEL-Descriptor v. 2.21 (National University of Singapore, Singapore, Singapore) [40]. The calculated descriptors were normalized to a 0–1 scale. In the descriptor reduction stage, 2264 with a variance equal to or close to zero were excluded. The pairwise correlation between descriptors was calculated, and 2360 with Pearson correlation coefficients > 0.90 were excluded. Then, the GreedyStepwise algorithm was used to select the most relevant combinations of descriptors for the QSAR model, and CfsSubsetEval was used to evaluate the selection of these descriptors, both of which were included in the Weka program (Waikato Environment for Knowledge Analysis) v. 3.7.0 (University of Waikato, Hamilton, New Zealand) [41]. The parameters used for the selection and evaluation of descriptors were those already established as standards by Weka. At this stage, 62 descriptors were selected for the development of the models. Finally, the Pearson correlation filter was applied to the selected descriptors, excluding those with a correlation coefficient > 0.60, providing a final set of 42 molecular descriptors. The correlation matrices can be viewed in Figures S3 and S4.

### 2.3. Machine Learning

After exploring the combination of 42 topological, structural, physicochemical, quantum–chemical two-dimensional chemical descriptors with the Random Forest (RF) algorithms and a decision tree driven by the AdaboostM1 method (J48/AdaboostM1), two classification models were generated to predict the probability that an alkaloid is potentially active in inhibiting SmTGR activities. Before prediction, the dataset was stratified into 70% for the training set and 30% for the test set. The chemical structures used for training and testing the RF and J48/AdaboostM1 models are available in Table S3 and Table S4, respectively.

### 2.4. Cross-Validation (CV)

A 10-fold cross-validation was used for internal validation of the models. This method is capable of detecting overfitting and outliers present in the training set [42]. The dataset was divided in a stratified manner into ten equally sized data subsets. Thus, one of the subsets, corresponding to 10% of the chemical structures in each dataset, was used as an external validation set. The other nine subsets, equivalent to 90% of the substances, were used to generate QSAR models. This procedure was performed ten times until each of the ten subsets was used as an external validation set.

### 2.5. External Validation (Ts)

The models were externally validated by evaluating the model’s ability to make predictions when applied to compounds outside the training set; in this case, the test set that was stratified was separated from the training set before the model was developed (30% of the dataset).

### 2.6. Model Evaluation

The performance of the classification models was evaluated using Cohen’s Kappa coefficient (k), which determines the agreement between the predicted values [43] and the corresponding area under the receiver operating characteristic curve ROC, which is a graph that shows the performance of a classification model and has two parameters: sensitivity, or true positive rate (tp%) on the Y axis, and specificity, or false positive rate (fp) on the X axis [44].

### 2.7. Applicability Domain (APD)

APD was used to flag substances in the test set for which the prediction may not be reliable. Similarity measures were used to define the applicability domain of the model based on Euclidean distances between all training and test substances. The distance of a test substance to its nearest neighbor in the training set is compared to the applicability domain limit (APD). If the similarity is beyond this threshold, the prediction is considered unreliable [45,46]. APD was calculated using the following formula:

APD = y+ Zσ(1)

where <y> is the average Euclidean distance of the 5 closest neighbors of the training set, σ is the standard deviation of the distances, and Z is an empirical cutoff value [45,46]. In this study, the Z value used was 0.5.

### 2.8. Construction of a Natural Product Alkaloid Database (AlcVS) for VS

A total of 844 alkaloids, originating from medicinal plants, were selected from scientific literature to build the virtual screening database. All alkaloids were drawn using the MarvinSketch program v. 19.22 (ChemAxon, Budapest, Hungary) [47]. The database contains the names of the substances, their codes in SMILES, classification according to the skeleton, family, species, and parts of the plant from which the alkaloid was extracted, as well as the articles that studied them.

### 2.9. Ligand-Based Virtual Screening (LBVS)

The workflow for virtual screening was carried out through the calculation of two-dimensional chemical descriptors for the alkaloid database, followed by the application of a node that normalizes the descriptors calculated for the alkaloids according to the normalization parameters provided in the model input (coming from the normalizer node applied to the database used in training). The generated and properly validated QSAR classification models were used for the virtual screening of a database composed of 844 alkaloids from medicinal plants to identify new alkaloids promising to inhibit SmTGR.

### 2.10. Consensus Analysis

To select alkaloids with the highest probabilities of activity (Patv), a virtual screening consensus analysis was performed using the following formula [8]:

(2) $$Pcm=\frac{\left(pRF\times ESPRF)+(pJ48/AdaboostM1\times ESPJ48/AdaboostM1\right)}{\left(ESPRF+ESPJ48/AdaboostM1\right)}$$

where Pcm is the combined probability between the models, pRF is the probability of the alkaloid being active in the RF model, ESPRF is the specificity of the RF model, pJ48/AdaboostM1 is the probability in the J48/AdaboostM1 model, and ESPJ48/AdaboostM1 is the specificity of the J48/AdaboostM1 model. In this equation, the specificity of each model is considered, which is inversely proportional to the false positive rate. Thus, each model’s Patv values are driven by a decrease in the false positive rate when considering specificity values. As a result, the models are less likely to select inactive molecules as active [8,34]. The equation also gives greater weight to the prediction of the model with a higher specificity value.

### 2.11. Activity Forecast Interpretation

The interpretation of the model results for the prediction of the alkaloids with the highest activities selected through the combined analysis was carried out using the Shapley Value Loop available on the Knime Analytics Platform v. 4.0.2 (KNIME AG, Zurich, Switzerland) [35]. Shapley Additive Explanations (SHAP) values are derived from game theory and are commonly used to explain the activity predictions of any machine learning algorithm, regardless of its complexity [48].

### 2.12. Molecular Docking and Structure-Based Virtual Screening (SBVS)

The crystallographic structure of the SmTGR enzyme was obtained from the Protein Data Bank (PDB) [49] platform code 6FP4, complexed with 1,8-naphthyridine-2-carboxylic acid (CAS: 215523-34-5). The structure was imported into the GOLD program v. 2022.1.0 (Cambridge Crystallographic Data Centre (CCDC), Cambridge, UK) [50] and prepared with the addition of hydrogen atoms and the removal of water molecules, as these elements could interfere with the docking process. Finally, the reference ligand that was previously crystallized with the structure was extracted. All coenzymes and cofactors originally present in the crystallographic structure, including FAD, were retained during both the redocking and molecular docking procedures in order to preserve the enzyme’s native conformation and the geometry of its active site.

Validation of the docking protocol was carried out after preparation. The structure was subjected to ligand extraction, and the coordinates of the co-crystallized ligand were used to define the center of the active site for redocking simulations. During this step, all scoring functions available in the GOLD program v. 2022.1.0 (Cambridge Crystallographic Data Centre (CCDC), Cambridge, UK)—ChemScore, CHEMPLP, ASP, and GoldScore—were tested. Each scoring function was evaluated using different radii for the docking site definition: 6 Å, 8 Å, 10 Å, 12 Å, and 14 Å.

Each redocking simulation was evaluated based on the root mean square deviation (RMSD) between the crystallographic and the predicted poses. A model was considered valid when the RMSD was below 2.0 Å [51]. Among the tested configurations, the ASP (Astex Statistical Potential) scoring function with a 6 Å radius produced the best result, with an RMSD of 0.89 Å and a score of 26.12. This configuration was therefore selected for subsequent docking simulations using the alkaloid database. Protein–ligand interactions were visualized and analyzed in PyMOL v.2.5 (Schrödinger, New York, NY, USA) [52] under a one-month free license.

### 2.13. Normalization of Score Values

The normalization of the scores obtained through molecular docking was performed to scale all values between 0 to 1, enabling comparison with Patv values obtained by LBVS [8,34,53]. In this study, alkaloids were classified as active when they presented structure-based probability values ≥ 0.60. Data normalization was conducted using the following formula:

(3) $$Ps=\frac{\left(Scores-min\right)}{\left(max-min\right)}$$

where Scores is the score obtained by molecular docking, min is the minimum score, and max is the maximum score of the dataset [53].

### 2.14. Combined Approach of LBVS and SBVS

The combination approach of SBVS and LBVS was carried out to identify potentially active alkaloids. This approach used the specificity rate of each QSAR model, thus seeking to minimize the probability of selecting false positive molecules [34]. Calculations were made with the following equation:

(4) $$Pc=\frac{\left(Ps+(1+ESPmed)\times Pcm\right)}{\left(2+ESPmed\right)}$$

where Pc is the combined probability, Ps is the structure-based probability, ESPmed is the average specificity rate of the models, and Pcm is the combined probability between models obtained by consensus analysis [8]. Next, the alkaloids most likely to inhibit SmTGR were selected.

## 3. Results and Discussion

### 3.1. Ligand-Based Virtual Screening (LBVS)

In the present study, a database comprising 356,643 substances tested in vitro at SmTGR (PubChem, AID: 485364) was selected, prepared, and balanced (as detailed in the Methods section). This process yielded a dataset containing 2504 substances, with 1325 active and 1179 inactive compounds against SmTGR, serving as the basis for developing QSAR models.

The dataset was modeled by exploring a combination of two-dimensional molecular descriptors encompassing topological, structural, physicochemical, and chemical–quantum characteristics. These descriptors were used to develop two QSAR classification models employing different machine learning algorithms.

The J48/AdaboostM1 model was constructed using 42 chemical descriptors, whereas the Random Forest (RF) model employed a subset of 31 descriptors. Despite this difference, both models yielded comparable performance metrics (Table 1). The QSAR models developed in this study demonstrated robust and predictive behavior, achieving accuracies ranging from 90.3% to 91.8% on the external test sets (Ts). Moreover, the models exhibited high sensitivity and specificity, indicating effective class differentiation with minimal rates of false positives and false negatives. In general, classification models with Cohen’s Kappa (κ) values between 0.60 and 0.80 are considered acceptable, while values between 0.80 and 1.00 indicate highly predictive performance [44]. Accordingly, based on the statistical results presented in Table 1, the constructed models achieved favorable prediction outcomes, with κ values exceeding 0.84 on the Ts set, thereby confirming their strong predictive capacity.

**Figure 1:**
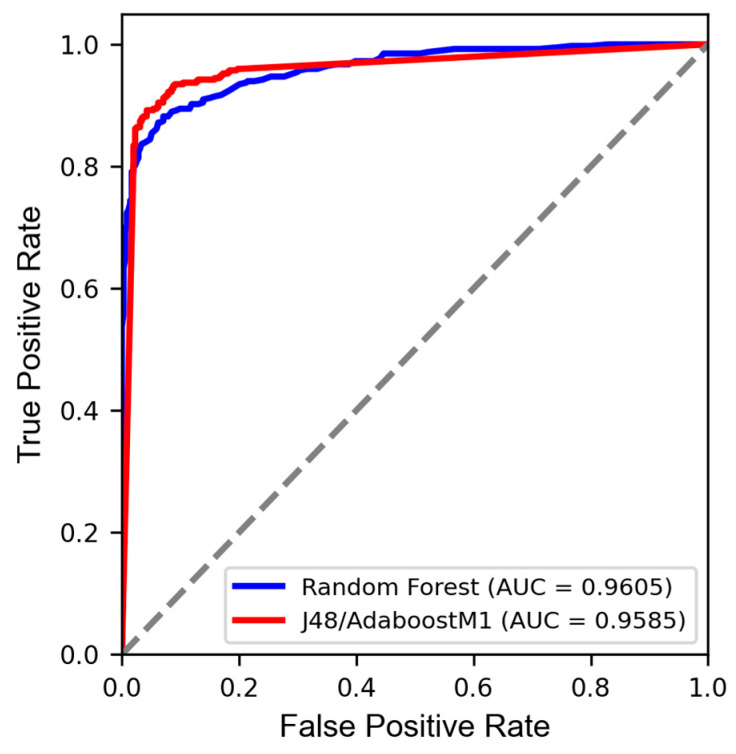
ROC curve for model J48/AdaboostM1 (blue line) and RF (red line). *y*-axis: true positive (tp) or sensitivity, and *x*-axis: false positive (fp) or 1-specificity. The closer to 1, the better the model. The light grey diagonal line in the diagram is the random line that represents the worst possible performance a model can achieve.

As depicted in Figure 1, both J48/AdaboostM1 and RF models exhibited minimal rates of false positives, leading to the high area under the ROC curve values of 0.96 for the Ts set. This indicates the models’ strong discriminatory power and robust predictive performance.

In general, a larger area under the ROC curve indicates a better-performing model [44]. Knowing that a perfect model has an area under the ROC curve equal to 1 [54], it is notable that the models demonstrate strong capability in distinguishing between active and inactive compounds, with good sensitivity.

**Figure 2:**
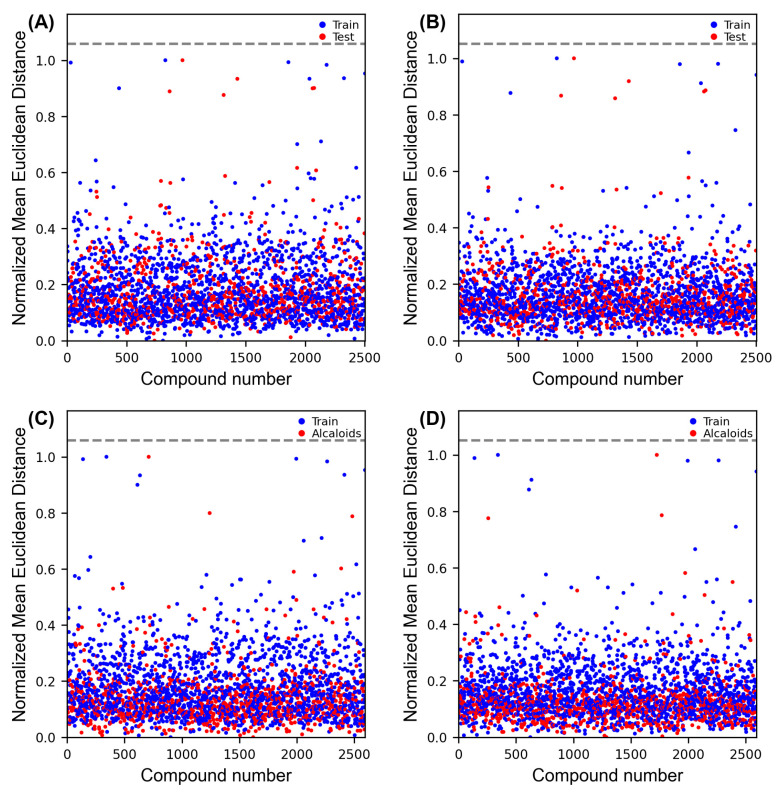
(**A**,**B**)—APD for J48/AdaboostM1 and RF models, respectively. (**C**,**D**)—APD for the alkaloids used for VS by the J48/AdaboostM1 and RF models, respectively, using the Euclidean distance approach (*y*-axis) and number of compounds present in the database (*x*-axis). The dashed grey line represents the threshold for the applicability domain.

All models in this study underwent applicability domain calculation (APD), confirming that predictions for all substances in the test set are 100% reliable. This is because all structures belong to the chemical space covered by the model (Figure 2A,B).

The models were then used for ligand-based virtual screening (LBVS). Of the 844 alkaloids, the RF model predicted 30 to be active, with the probability of activity (Patv) ranging from 51.2 to 88.8%. Meanwhile, the J48/AdaboostM1 model identified 24 alkaloids with Patv between 79.3% and 100%.

Interestingly, both models concurred on the prediction of 13 alkaloids, resulting in a total of 39 alkaloids being designated as active by the models (refer to Table S1). From Figure 2C,D, it is evident that all alkaloids in the database are in the chemical space covered by the constructed models. This confirms the reliability of their predictions for the 39 selected alkaloids.

### 3.2. Consensus Analysis

To identify alkaloids with the highest probability of being active against *S. mansoni*, a consensus analysis of LBVS predictions was performed, resulting in the selection of 23 alkaloids with Patv values ranging from 56.4 to 89.2%, as predicted by the developed models (Table 2). This consensus approach enhanced the likelihood of correctly identifying active compounds, as alkaloids selected by both machine learning algorithms are more likely to exhibit true biological activity.

### 3.3. Interpretation of Descriptors with the Greatest Contributions to Alkaloid Inhibition Activity

**Figure 3:**
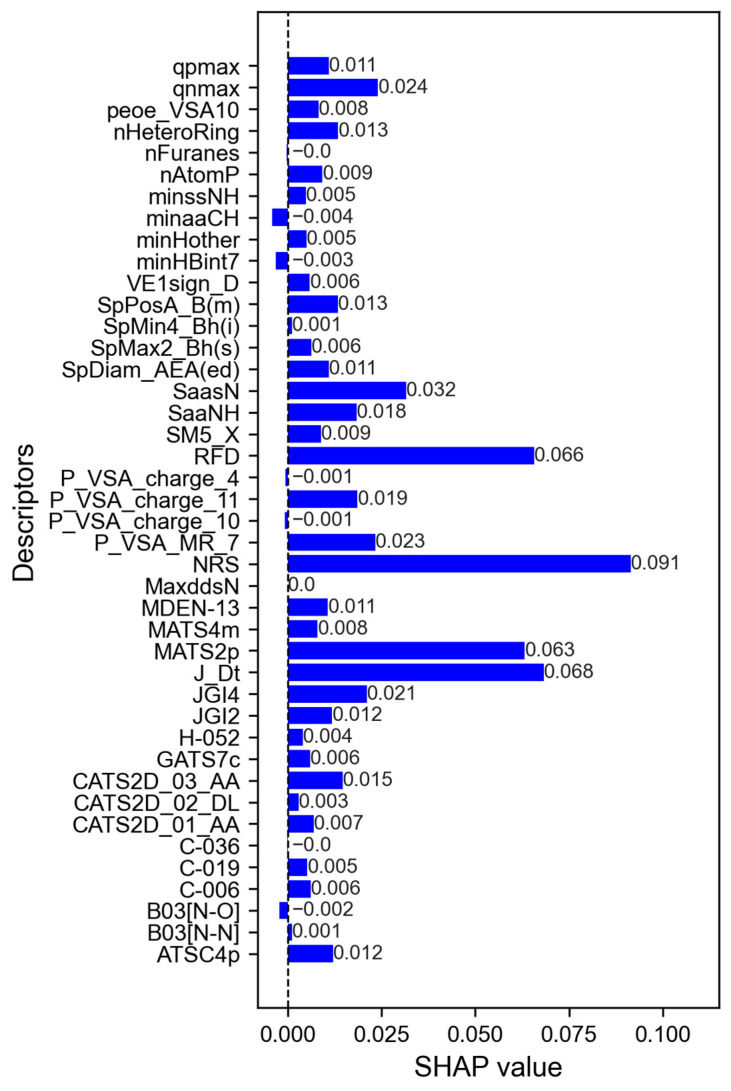
Average values of the contributions of the descriptors to the activity of the alkaloids. *y*-axis: Descriptors used to build the models. *x*-axis: mean SHAP values (descriptor contributions). Descriptors with positive values contribute to the activity prediction, while descriptors with negative values contribute negatively to the activity.

Both J48/AdaboostM1 and RF models shared the four most relevant descriptors for alkaloid activity (Figure 3). Notably, the number of ring systems (NRS) was the most significant descriptor influencing alkaloid activity. These findings align with existing knowledge that the number of aromatic rings in a structure directly contributes to increasing the hydrophobicity of the molecule [55], as aromatic rings play a considerable role in the properties of molecules, such as molecular reactivity, polarity, or lipophilicity [56]. Hydrophobicity is a way of evaluating the preference of a compound to have a greater affinity in a hydrophobic environment compared to an aqueous one. Descriptors that estimate the hydrophobicity of a molecule are fundamental in studies of structure–property relationships (SPR) [57].

The second descriptor with significant contributions to alkaloid activity is the J_Dt, or Balaban-type index of the deviation matrix, which is a topological descriptor providing insights into atom distances. The Balaban index considers factors such as the presence of multiple bonds and heteroatoms [58] and has been effectively used in several QSAR models [59] as it correlates well with a variety of physicochemical properties of the molecule [60].

The third most significant descriptor is the polarizability descriptor, MATS2p, derived from the autocorrelation of the Moran topological structure (MATS). This descriptor addresses the topology of the structure or its components in conjunction with a physicochemical property—in this case, polarizability. The numerical designation ‘2’ denotes the maximum distance on the graph [61].

The fourth descriptor with high contributions also belongs to the class of ring descriptors, the RFD, representing the ring fusion density. Ring fusion turns the molecule rigid with increased π stacking due to the polarization of the electron clouds. Ring fusion leads to an increase in the level of the lowest-energy unoccupied molecular orbital (LUMO), making the molecule electronegative and enhancing its electron-attracting power, which makes the molecule more electrophilic [62,63]. It is also known that TGR is responsible for maintaining the redox state of parasites but also provides a good nucleophilic binding site for inhibitors [64].

These results indicate that the chemical properties of hydrophobicity, polarizability, and electronegativity are important for the inhibition activity of alkaloids against SmTGR. In Figures S1 and S2, it is possible to check the descriptors with the greatest contributions to the activity of the alkaloids predicted by the models individually.

In contrast, the atom-type minimum electrotopological state descriptors (minHBint7 and minaaCH) [65,66] showed negative contributions to alkaloid activity. The minHBint7 descriptor reveals that a decrease in hydrogen bond potentials of path length 7 is favorable for the inhibitory activity of alkaloids. The descriptor minaaCH denotes the minimum value of the absence of the electrotopological state of C-H fractions in an aromatic molecule [66]. The negative contribution of this descriptor may indicate that the presence of aromatic rings increases the inhibition activity of alkaloids against TGR. The descriptor B03[N-O] presented a negative contribution to the prediction of alkaloid activity, indicating that the absence of [N-O] bonds at topological distance 3 disfavors the alkaloid inhibition activity and vice versa.

### 3.4. Structure-Based Virtual Screening (SBVS)

SBVS was performed targeting a non-catalytic regulatory site of SmTGR known as the “doorstop pocket” [67].

Although this pocket is not part of the enzyme’s main electron transfer pathway, its proximity to the NADPH-binding site and low homology with human TrxR make it an attractive target for selective inhibition. Previous studies have highlighted this cavity as a promising region for designing competitive inhibitors that do not depend on the enzyme’s catalytic activity. This rationale, combined with the structural quality and docking validation metrics (RMSD < 2 Å) of the 6FP4 crystal structure, supported its selection as the primary target for docking simulations.

In the process of validating the docking protocol, through redocking of the ligand co-crystallized with SmTGR, the ASP (Astex statistical potential) scoring function presented the best model within a radius of 6 Å with an RMSD of 0.89 and a score of 26.12. After docking validation, molecular docking was performed for the 23 alkaloids predicted to be active through consensus analysis.

All alkaloids scored higher than the PDB ligand, suggesting stronger interactions between alkaloids and the enzyme’s active site residues (Table S2).

### 3.5. The Combined Approach

After normalizing the score values obtained by molecular docking, a structure- and ligand-based virtual screening combination approach was carried out to verify potentially active molecules. Alkaloids with Patv values greater than 60% were classified as active. The screening resulted in the selection of 14 alkaloids containing Patv values ranging from 61.2 to 85.2%, analyzed by visual inspection at the protein binding site (Table 3).

**Figure 4:**
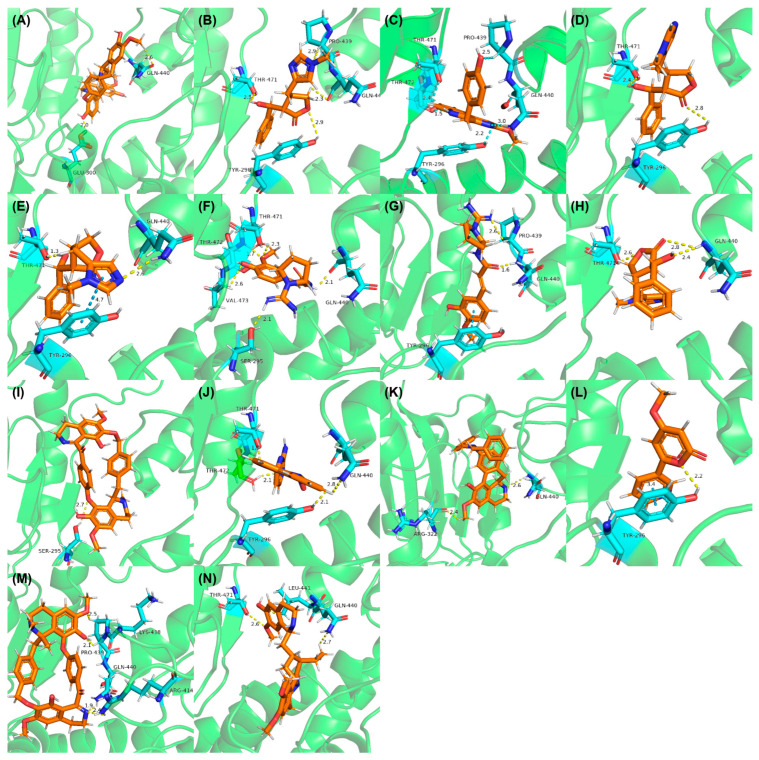
Interaction of alkaloids (orange, carbon atoms) with amino acids in the “doorstop pocket” site. (**A**) Lindoldhamine, (**B**) Epiisopiloturine, (**C**) Daibucarbolines, (**D**) Epiisopilosine, (**E**) Pilosine, (**F**) Isocernumedine, (**G**) Cernumedine, (**H**) Isopilosine, (**I**) Epi-des-7′-*O*-methylroraimine, (**J**) N-Hydroxyannomontine, (**K**) Variabiline, (**L**) Anibine, (**M**) Des-7′-*O*-methylroraimine, and (**N**) Emetine, where the green ribbon denotes the protein backbone, yellow dashed lines indicate hydrogen bonds, and cyan dashed lines represent pi-pi stacking/hydrophobic interactions (E, G, L).

The visual inspection of the molecular docking poses of the selected chemical structures (Figure 4) indicated that the hydroxyl, amino, methoxy, carbonyl, and ether groups, as well as the oxygen of the pyran and furan ring of alkaloids, could interact in the binding site by hydrogen bonds with the amino acids THR471, THR472, PRO439, ARG414, ARG322, LYS438, LEU441, SER295, TYR296, VAL473, GLU300, and GLN440. Some alkaloids performed π-stacking interactions with TYR296, which is a type of dipole–dipole interaction between aromatic rings (Figure 4E,G,L).

The π-electron clouds of the alkaloids and TYR296 align in a parallel orientation, due to the polarization of the electronic clouds, as can be seen in Anibine (Figure 4L). In another type of interaction, the aromatic rings can also reach a 90-degree plane—in this case, called T-stacking—which can be visualized with the Pilosine alkaloid (Figure 4E). The interaction energy when π-systems are in a T-shape is more favorable than when the systems are stacked. These interactions, despite their relatively weak nature, are important for the stability of protein–ligand complex formation [68].

Of those alkaloids classified as potentially active, four were imidazole alkaloids epiisopiloturine, epiisopilosine, pilosine, and isopilosine (Figure 4B,D,E,H), respectively, isolated from *Pilocarpus microphyllus* (Rutaceae), a plant species already identified as active against helminths and with a known schistosomicidal activity according to Rocha et al. [24].

Rocha et al. [24] also carried out molecular docking studies, and it was found that the studied alkaloids interact through hydrophobic and hydrogen regions with SmTGR [26]. Thus, the results found in this research are in accordance with reports in the literature showing that the set of modeling techniques used was efficient for building prediction models. In another study carried out by Guimarães et al. [23], epiisopilosine revealed in vivo activity against adult and juvenile *S. mansoni*. These results corroborate the studies carried out by Angelucci et al. [18], who stated that inhibition of SmTGR is also effective against immature forms of the parasite.

In addition to the alkaloids mentioned above, the present study selected 10 alkaloids as potentially active, and which have not yet been reported in the literature as SmTGR inhibitors. The Lindoldhamine and Daibucarboline A, (Figure 4A and Figure 4C, respectively) alkaloids presented Pc values equal to 85.2 and 75.8%, respectively, higher than the Pc obtained for the imidazole alkaloids (Table 3).

Lindoldhamine, a bisbenzylisoquinoline alkaloid isolated from the leaves of *Laurus nobilis* (Lauraceae), popularly known as “bay leaf”, and also from the leaves of *Triclisia sacleuxii* (Pierre) Diels (Menispermaceae), has several biological activities reported in the literature [69,70]. Of these, it is possible to mention the trypanocidal activity by inhibiting the trypanothione reductase enzyme [70]. Like the *S. mansoni* TGR, trypanothione reductase is also an oxidoreductase, and both belong to the GR superfamily of dimeric flavoenzymes, characterized by having a highly similar structure and reaction mechanisms [71]. Therefore, the Lindoldhamine alkaloid has great potential to also inhibit SmTGR.

The indole alkaloid Daibucarboline A is isolated from the roots of *Neolitsea daibuensis* (Lauraceae) [72,73]. Another indole alkaloid, N-Hydroxyannomontine (Figure 4J), extracted from the bark of *Annona foetida* (Annonaceae), presented in vitro antiparasitic activity against *Leishmania braziliensis* and *Leishmania guyanensis* [74,75].

The alkaloids Isocernumidine and cernumidine (Figure 4F and Figure 4G, respectively), isolated from the leaves of *Solanum cernuum Vell*. (Solanaceae), a Brazilian medicinal plant popularly known as “panacea” [76]. The bisbenzylisoquinoline alkaloids epi-des-7’-*O*-methylroraimine and des-7′-*O*-methylroraimine (Figure 4I and Figure 4M, respectively), were isolated from the rhizome of *Cissampelos sympodialis Eichl*. (Menispermaceae) [77], popularly known as “Milona” [78]. Variabiline (Figure 4K) is an aporphine alkaloid extracted from the plant *Ocotea variabilis* (currently accepted as *Ocotea lancifolia*, Lauraceae) [79]. The anibine alkaloid of the pyridine class (Figure 4L) is extracted from the stems of *Aniba rosaeodora* and *Aniba fragrans* (Lauraceae) [80]. Emetine (Figure 4N) is a tetrahydroisoquinoline alkaloid found predominantly in the roots of the plant *Carapichea ipecacuanha (Brot.)* [81]. In Table S5, it is possible to visualize the geographic distribution of the plant species where the alkaloids can be found.

The previously mentioned alkaloids have not yet been reported in the literature for their antiparasitic activity, representing a promising opportunity for further research in the search for compounds with schistosomicidal activity.

## 4. Conclusions

This study employed a combination of computational approaches and a chemically diverse alkaloid database to develop QSAR models aimed at predicting promising inhibitors of SmTGR, an essential enzyme involved in the reactive oxygen species (ROS) detoxification system of *S. mansoni*. Notably, the initial chemical element filtering and the Tanimoto similarity-based balancing strategy facilitated the selection of structurally consistent compounds and the development of calibrated models capable of effectively distinguishing between active and inactive alkaloids. By applying a combination of virtual screening methods to a database comprising 844 alkaloids derived from various plant species, seven compounds not previously reported as SmTGR inhibitors were identified, each exhibiting a predicted probability of activity (Patv) above 70%. These alkaloids represent promising candidates for the development of new drugs for the treatment of schistosomiasis, a neglected tropical disease of high prevalence and public health relevance. Moreover, the developed models may be applied to identify additional alkaloids beyond the current database, as well as to predict the activity of other nitrogen-containing compound classes that fall within the same chemical space. Intermolecular interactions between the selected alkaloids and SmTGR residues were explored through molecular docking, revealing hydrogen bonds and π–π stacking interactions involving aromatic ring systems. These findings align with the interpretation of the predictive models, which identified the RFD and NRS descriptors as key contributors to biological activity.

## Figures and Tables

**Table 1:** Statistical results of the classificatory QSAR models.

Models		Ac (%)	κ	ROC Curve	SE	SP	Coverage (%)
RF	Tr	100	1	1	1	1	
	CV	90.4	0.81	0.96	0.88	0.94	
	Ts	90.3	0.81	0.96	0.87	0.94	100%
J48/AdaboostM1	Tr	100	1	1	1	1	
	CV	91.9	0.84	0.96	0.89	0.95	
	Ts	91.8	0.84	0.96	0.91	0.93	100%

RF: Random forest; J48/AdaboostM1: Decision Tree combined with the AdaboostM1 method; κ: coefficient κ de Cohen; ROC curve: receiver operating characteristic curve; Tr: training set; CV: cross-validation; Ts: test set; Coverage: percentage of compounds in the validation set within the applicability domain (APD); Ac: accuracy; SE: sensitivity; SP: specificity.

**Table 2:** Potentially active alkaloids from consensus analysis.

Alkaloids	Pcm (%)	Alkaloids	Pcm (%)
Episiopiloturine	89.2	Lindoldhamine	77.5
N-Hydroxyannomontine	87.2	Catuabine I	72.8
Epiisopilosine	86.8	7α-hydroxycatuabine H	69.1
Isopilosine	86.8	7β-hydroxycatuabine H	69.1
Pilosine	86.8	Cis-*N*-Oxycodamine	68.3
Daibucarboline A	83.5	Cephaeline	67.9
Anibine	82.7	Emetine	67.9
Des-7-*O*-methylroraimine	81.5	Vaccinine B	67.5
Epi-des-7-*O*-methylroraimine	81.5	Siamine	65.5
Cernumidine	79.9	Tueiaoine	63.1
Isocernumidine	79.5	Alstomicine	56.4
Variabiline	77.5		

Pcm: Patv obtained from consensus analysis.

**Table 3:** Results obtained for the 14 alkaloids from structure- and ligand-based VS.

Structures	Alkaloids	Pcm (%)	Ps (%)	Pc (%)
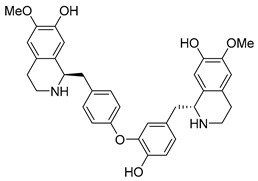	Lindoldhamine	77.52	100	85.18
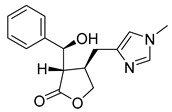	Episiopiloturine	89.17	51.26	76.25
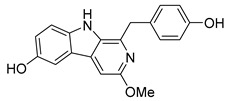	Daibucarboline A	83.55	60.85	75.81
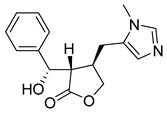	Epiisopilosine	86.76	50.16	74.29
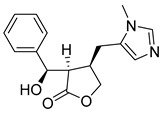	Pilosine	86.76	44.96	72.52
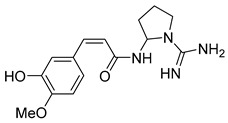	Isocernumidine	79.53	50.06	69.49
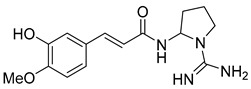	Cernumidine	79.94	46.16	68.43
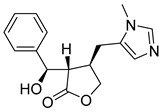	Isopilosine	86.76	31.76	68.02
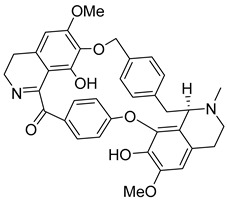	Epi-des-7-*O*-methylroraimine	81.54	39.86	67.34
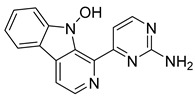	N-Hydroxyannomontine	87.16	18.66	63.82
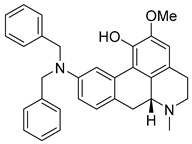	Variabiline	77.53	36.15	63.43
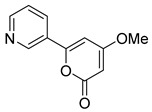	Anibine	82.74	25.86	63.36
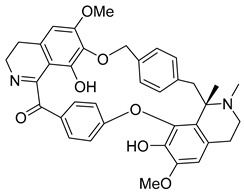	Des-7-*O*-methylroraimine	81.54	26.15	62.67
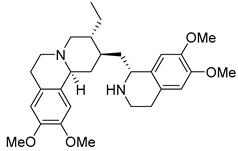	Emetine	48.39	25.86	61.25

Pcm: combined probability between models in ligand-based VS; Ps: probability value in structure-based VS; Pc: probability of combined approach.

## Data Availability

The original contributions presented in this study are included in the article/Supplementary Materials. Further inquiries can be directed to the corresponding authors.

## Supplementary Materials

The following supporting information can be downloaded at: https://www.mdpi.com/article/10.3390/pathogens14060591/s1, Figure S1: Descriptors with greatest contributions to the activity of alkaloids predicted by the RF model; Figure S2: Descriptors with greatest contributions to the activity of alkaloids predicted by the AdaboostM1 model; Table S1: Alkaloids predicted to be active by the RF model and the J48/AdaboostM1 model; Figure S3: Correlation matrix of descriptors selected to build the J48/AdaboostM1 model; Figure S4: Correlation matrix of descriptors selected for building the RF model; Table S2: Interaction of alkaloids with SmTGR; Table S3: Structures of the train and test set and descriptors used to build the Random forest model; Table S4: Structures of the train and test set and descriptors used to build the J48/AdaboostM1 model. Table S5: Geographical distribution of plant species [82,83].

## Abbreviations

PZQ	Praziquantel
SmTGR	Thioredoxin Glutathione Reductase from *Schistosoma mansoni*
VS	Virtual Screening
EROS	Oxygen-reactive species
ROC	Receiver Operating Characteristic Curve
SBVS	Structure-Based Virtual Screening
LBVS	Ligand-Based Virtual Screening
Trx	Thioredoxin
TrxR	Thioredoxin Reductase
Grx	Glutaredoxin
GR	GSH Reductase
GSH	Glutathione
QSAR	Quantitative Structure-Activity Relationship
RF	Randon Forest
TSs	External test
CV	Cross-validation
Tr	Train
Patv	Activity probability percentage
ASP	Astex Statistical Potential
APD	Applicability Domain
