# Supplementary material for: Novel Antischistosomal Drug Targets: Identification of Alkaloid Inhibitors of SmTGR via Integrated In Silico Methods
Source: Pathogens. 2025 Jun 15;14(6):591. doi: 10.3390/pathogens14060591 (PMC12196247; doi:10.3390/pathogens14060591)
Supplement: Supplementary file 1 [file pathogens-14-00591-s001.zip › Supplementary Information.pdf]

Supplementary Information

Novel Antischistosomal Drug Targets: Identification of Alkaloid Inhibitors of SmTGR via Integrated In Silico Methods

Valéria V. M. Paixão [1], Yria J. A. Santos [1], Adriana O. Fernandes [2], Elaine S. Conceição [1], Ricardo P. Rodrigues [3], Daniela A. Chagas-Paula [4], Silvio S. Dolabella [5,6,*] and Tiago B. Oliveira [1,2,7,*]

1 Posgraduate Program in Chemistry—PPGQ, Federal University of Sergipe, Av. Marcelo Deda Chagas, s/n, Bairro Rosa Elze, São Cristóvão 49107-230, SE, Brazil; valeriavieiramp1994@gmail.com (V.V.M.P.);
yria.a.santos@gmail.com (Y.J.A.S.); elainesantosconceicao96@gmail.com (E.S.C.)

2 Postgraduate Program in Biotechnology—PROBIO, Federal University of Sergipe, Av. Marcelo Deda Chagas, s/n, Bairro Rosa Elze, São Cristóvão 49107-230, SE, Brazil; ad00fernandes@gmail.com

3 Faculty of Pharmaceutical Sciences, University of Campinas, Rua Cândido Portinari, 200, Cidade Universitária, Campinas 13083-871, SP, Brazil; rrodrigues@fcf.unicamp.br

4 Chemistry Institute, Federal University of Alfenas, Rua Gabriel Monteiro da Silva, Alfenas 37130-001, MG, Brazil; daniela.chagas@unifal-mg.edu.br

5 Postgraduate Program in Parasite Biology, Federal University of Sergipe, Av. Marcelo Deda Chagas, s/n, Bairro Rosa Elze, São Cristóvão 49107-230, SE, Brazil

6 Postgraduate Program in Pharmaceutical Sciences, Federal University of Sergipe, Av. Marcelo Deda Chagas, s/n, Bairro Rosa Elze, São Cristóvão 49107-230, SE, Brazil

7 Pharmacy Department, Federal University of Sergipe, Av. Marcelo Deda Chagas, s/n, Bairro Rosa Elze,
São Cristóvão 49107-230, SE, Brazil

* Correspondence: dolabellaufs@gmail.com (S.S.D.); tiago.branquinho@ufs.br (T.B.O.)

Figure S1: Descriptors with greatest contributions to the activity of alkaloids predicted by the RF model.

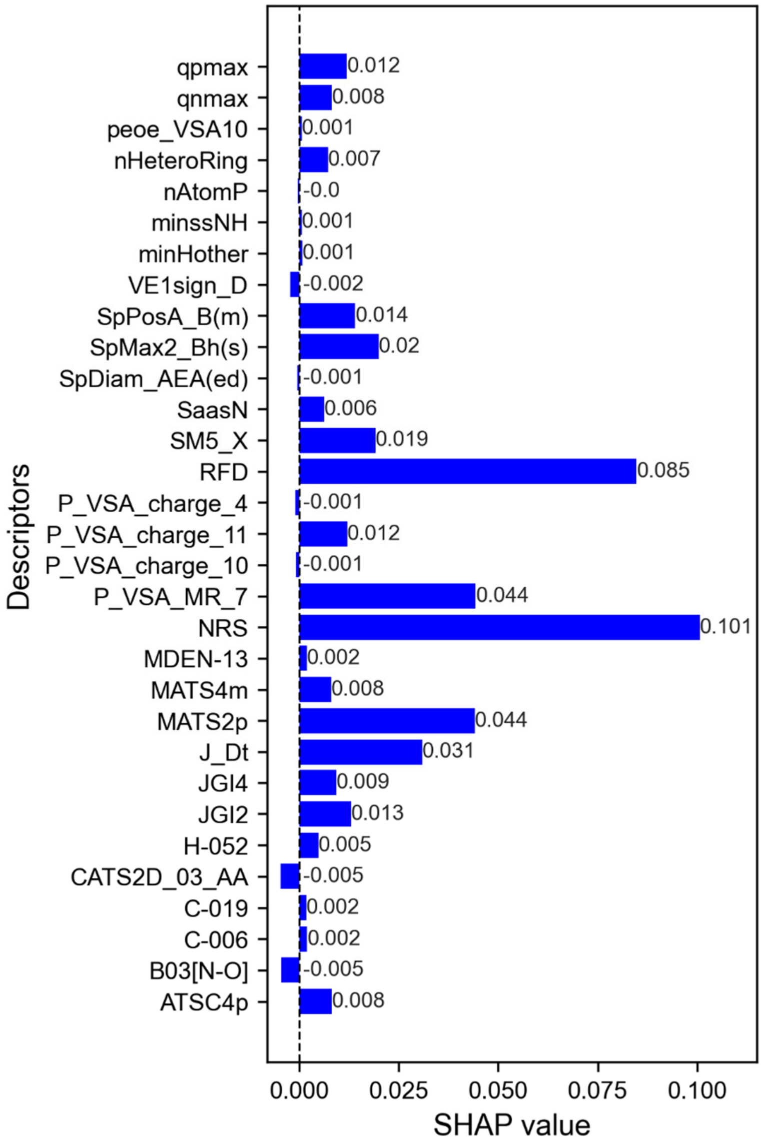

Figure S2: Descriptors with greatest contributions to the activity of alkaloids predicted by the AdaboostM1 model.

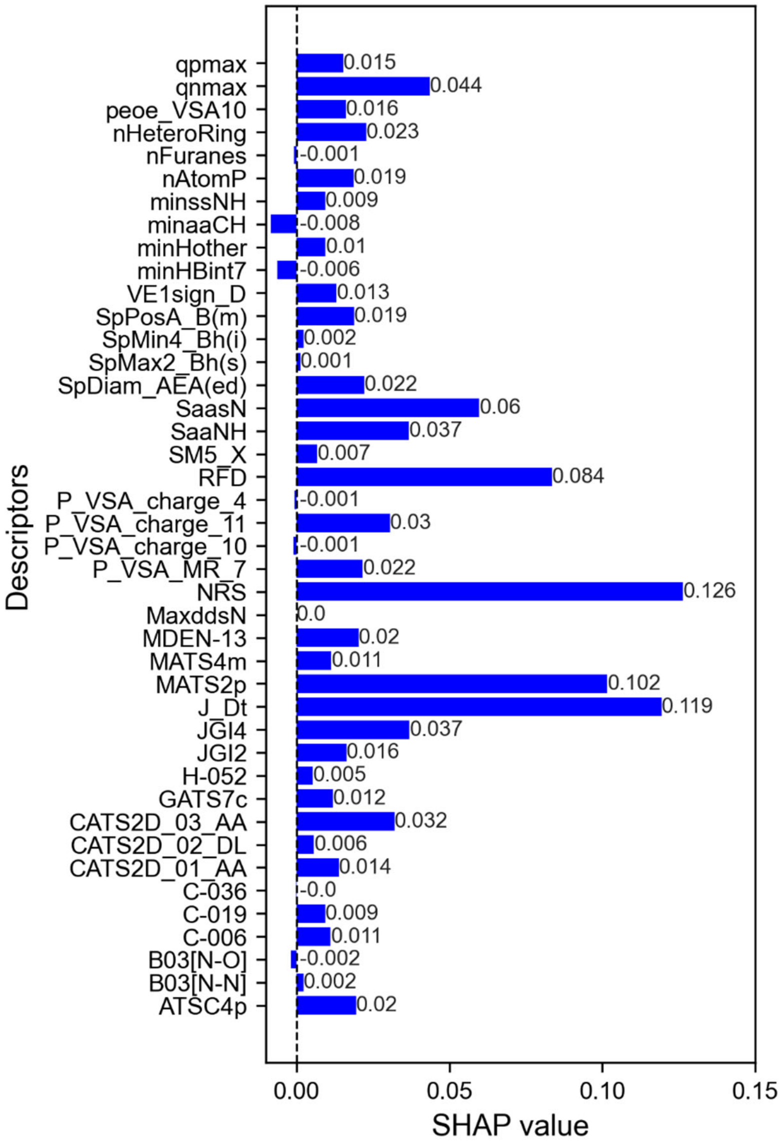

Table S1: Alkaloids predicted to be active by the RF model and the J48/AdaboostM1 model.

Alkaloids (RF)	p(Activity) %	Alkaloids (J48/AdaboostM1)	p(Activity) %
6_8_didec_(1Z)_eni_5_7_dimethyl2_3_dihydro_1H_indolizidine	61,6	S-variabiline	100
Braquicerina	56,0	7α-hydroxycatuabine H	100
Anocerina A	67,2	7β-hydroxycatuabine H	100
Anocerina B	61,6	Cis-N-Oxycodamine	100
7-O-senecioylretronecine	60,8	Emetine	100
9-O-senecioylretronecine	56,8	Siamine	100
Daibucarbolines B	72,0	Alstomicine	79,3
Daibucarbolines C	66,4	Catuabine I	97,7
Cecilin	56,8	Cephaeline	100
Clorgilina	63,2		
Daibucarbolines A*	67,2		100
Anibine*	65,6		100
Cernumidine*	60,0		100
Des-7-O-methylroraimine*	63,2		100
Discareno C	58,4		
Epi-des-7-O-methylroraimine*	63,2		100
Epiisopilosine*	73,6		100
Episiopiloturine*	78,4		100
Indicumine B	76,0		
Indolizidina	60,8		
Isocernumidine*	59,2		100
Isopilosine*	73,6		100
Lindoldhamine*	55,2		100
Lyaloside	0.832		
N-Hydroxyannomontine*	0.744		100
Pauridianthoside	0.888		
Pilosine*	0.736		100
Psicolatina	0.552		
Robustina	0.512		
R-variabiline*	0.552		100

* Alkaloids predicted to be active by both models.

Figure S3: Correlation matrix of descriptors selected to build the J48/AdaboostM1 model.

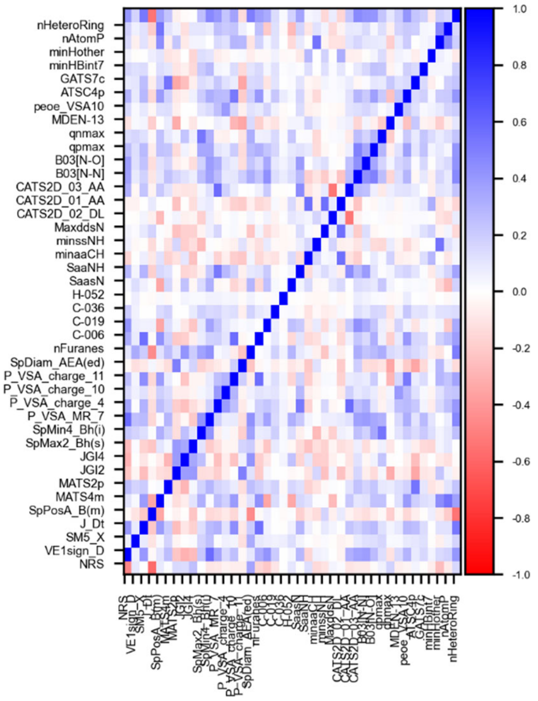

Figure S4: Correlation matrix of descriptors selected for building the RF model.

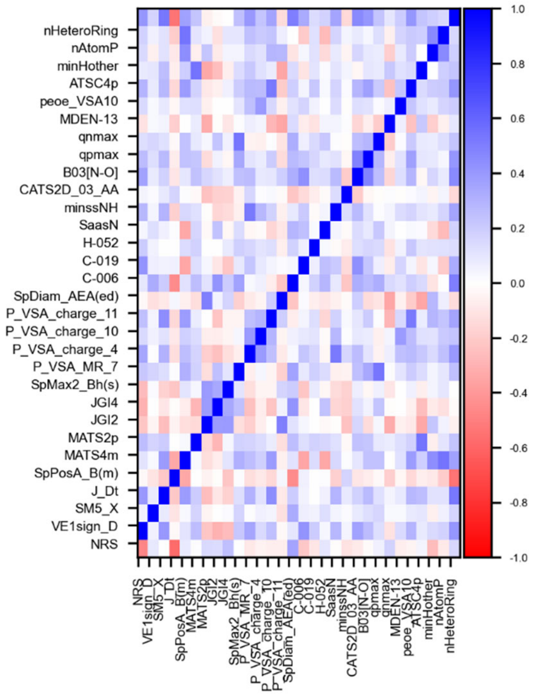

Table S2: Interaction of alkaloids with SmTGR.

Alcaloides	Score
7α-hydroxycatuabine-H	42,54
7β-hydroxycatuabine-H	50,68
Alstomicine	57,79
Anibine	50,55
Daiucarbolines A	61,39
Catuabine I	51,39
Cephaeline	55,14
Cernumidine	56,84
Cis-N-Oxycodamine	52,36
Des-7-O-methylroraimine	50,64
Emetine	57,53
Epi-des-7-O-methylroraimine	54,89
Epiisopilosine	58,08
Episiopiloturine	58,42
Isocernumidine	58,05
Isopilosine	52,38
Lindoldhamine	73,52
N-Hydroxyannomontine	48,32
Pilosine	56,47
R-variabiline	53,74
Siamine	49,62
Tueiaoine	56,60
Vaccinine B	45,99

Table S5: Geographical distribution of plant species

Specie	Distribution
Pilocarpus microphyllus (Rutaceae)	Native to: north and northeast Brazil[a]
Laurus nobilis (Lauraceae)	Native to:<br>Albania, Algeria, Corse, Cyprus, East Aegean Is., France, Greece, Italy, Kriti, Lebanon-Syria, Libya, Morocco, NW. Balkan Pen., Palestine, Sardegna, Sicilia, Tunisia, Türkey, Türkey-in-Europe[a]<br>Introduced into:<br>Azores, Baleares, Great Britain, Ireland, Korea, Krym, North Caucasus, Portugal, Spain, Transcaucasus, Vietnam[a]
Neolitsea daibuensis (Lauraceae)	Native to:<br>Taiwan[a]
Annona foetida (Annonaceae)	Native to:<br>Bolivia, Brazil North, Brazil West-Central, Colombia, French Guiana, Peru e Suriname[a]

Solanum cernuum Vell. (Solanaceae)	Native to:<br><br>Brazil Northeast, Brazil Southeast, Brazil West-Central[a]
Cissampelos sympodialis Eichl. (Menispermaceae)	Native to:<br><br>Brazil North, Brazil Northeast, Brazil Southeast[a]
Ocotea variabilis (Lauraceae)	Found in:<br><br>Southern America Brazil Brazil North, Tocantins, Brazil Northeast, Bahia, Brazil South, Paraní, Rio Grande do Sul, Santa Catarina, Brazil Southeast, Espirito Santo, Minas Gerais, Rio de Janeiro, São Paulo, Brazil West-Central, Brazilia Distrito Federal, Goiás, Mato Grosso do Sul, Southern South America Argentina Northeast, Paraguay and Western South America Bolivia[b]
Aniba rosaeodora (Lauraceae)	Found in:<br><br>Southern America Brazil Amapí, Amazonas, Parí[b]
Carapichea ipecacuanha (Brot.)	Found in:<br><br>Southern America: Brazil North, Rondônia, Brazil Northeast, Bahia, Pernambuco, Brazil South, Brazil Southeast, Espirito Santo, Minas Gerais, Rio de Janeiro, São Paulo, Brazil West-Central, Goiás, Mato Grosso, Central America Costa Rica, Nicaragua, Panamá, Western South America Colombia[b]

[a] POWO (2025). "Plants of the World Online. Facilitated by the Royal Bo-tanic Gar-dens, Kew. Published on the In-ternet; https://powo.science.kew.org/ Retrieved 03 June 2025."

[b]WFO (2025): Cara-pichea ip-ecacuanha (Brot.) L.Andersson. Pub-lished on the Inter-net; http://www.worldflo-raon-line.org/taxon/wfo-0000336046. Accessed on: 03 Jun 2025
